# Estimation of the Number of Patients With Mitochondrial Diseases: A Descriptive Study Using a Nationwide Database in Japan

**DOI:** 10.2188/jea.JE20200577

**Published:** 2023-02-05

**Authors:** Koki Ibayashi, Yoshihisa Fujino, Masakazu Mimaki, Kenji Fujimoto, Shinya Matsuda, Yu-ichi Goto

**Affiliations:** 1 Department of Environmental Epidemiology, Institute of Industrial Ecological Sciences, University of Occupational and Environmental Health, Japan, Fukuoka, Japan; 2 Department of Pediatrics, Teikyo University School of Medicine, Tokyo, Japan; 3 Department of Public Health, University of Occupational and Environmental Health, Japan, Fukuoka, Japan; 4 Department of Mental Retardation and Birth Defect Research, National Center of Neurology and Psychiatry (NCNP), Tokyo, Japan

**Keywords:** insurance claim review, Japan, medical records, mitochondrial diseases, prevalence

## Abstract

**Background:**

To provide a better healthcare system for patients with mitochondrial diseases, it is important to understand the basic epidemiology of these conditions, including the number of patients affected. However, little information about them has appeared in Japan to date.

**Methods:**

To gather data of patients with mitochondrial diseases, we estimated the number of patients with mitochondrial diseases from April 2018 through March 2019 using a national Japanese health care claims database, the National Database (NDB). Further, we calculated the prevalence of patients, and sex ratio, age class, and geographical distribution.

**Results:**

From April 2018 through March 2019, the number of patients with mitochondrial diseases was 3,629, and the prevalence was 2.9 (95% confidence interval [CI], 2.8–3.0) per 100,000 general population. The ratio of females and males was 53 to 47, and the most frequent age class was 40–49 years old. Tokyo had the greatest number of patients with mitochondrial diseases, at 477, whereas Yamanashi had the fewest, at 13. Kagoshima had the highest prevalence of patients with mitochondrial diseases, 8.4 (95% CI, 7.1–10.0) per 100,000 population, whereas Yamanashi had the lowest, 1.6 (95% CI, 0.8–2.7).

**Conclusion:**

The number of patients with mitochondrial diseases estimated by this study, 3,269, was more than double that indicated by the Japanese government. This result may imply that about half of all patients are overlooked for reasons such as low severity of illness, suggesting that the Japanese healthcare system needs to provide additional support for these patients.

## INTRODUCTION

Mitochondrial diseases are caused by nuclear or mitochondrial DNA mutations, and patients vary in age of onset, sex, race, affected organ, severity, and prognosis.^1^^–^^3^ While mitochondrial diseases were long thought to be rare, recent reports^4^^–^^19^ have suggested that the prevalence of patients in the general population is higher than originally thought. For example, prevalence is 23 per 100,000 general adult population in the northeast of England^6^ and 5.7 per 100,000 general adult population in Spain.^15^ While there are few reports of mitochondrial diseases in Japan,^17^^,^^18^ a questionnaire study reported a prevalence of 0.18 per 100,000 general population.^18^ However, this study was limited to mitochondrial myopathy, encephalopathy, lactic acidosis, and stroke-like episodes (MELAS), which comprise only a proportion of mitochondrial diseases. Thus, the prevalence of overall mitochondrial diseases in Japan remains unclear.

In Japan, mitochondrial disease was recognized as an intractable disease under the designation of the Japanese Ministry of Health, Labor, and Welfare (MHLW) in 2009, which brought about various improvements in the treatment of these conditions. However, several studies have identified issues related to the treatment of mitochondrial diseases.^20^^,^^21^ First, diagnosis requires a high degree of expertise. Definitive diagnosis requires an integrated approach comprising imaging, pathology, electrophysiology, genetics, and biochemical examinations, in addition to the main clinical manifestation, and the number of well-trained clinicians and hospitals equipped to perform these tests currently appears insufficient for patient needs. Second, recent developments in treatment have increased the survival of patients; in particular, the establishment of a system for transitional care from childhood to adulthood is suggested to be a major factor.^21^ Third, the prevalence of some DNA mutations that cause mitochondrial diseases differs by geography.^19^ This can make it difficult for patients to gain equal access to medical care, albeit that no reports of geography-related problems have appeared in Japan to date.

Accordingly, to ensure that policy makers make informed decisions and patients and their caregivers receive the best possible care, it is essential to gather basic epidemiological information on mitochondrial diseases, including the number of patients and their distribution by age, sex, and geographic location.

Here, we used a nationwide Japanese health claims database to estimate the number and other epidemiological parameters of patients with mitochondrial diseases.

## METHODS

### Data source and patient selection

We used the National Database (NDB), run by the Japanese MHLW,^22^^,^^23^ to extract data on patients with mitochondrial diseases. The NDB contains information on almost all healthcare claims made from April 2009 in Japan, including patient sex; age class; numerical diagnosis code,^24^ which is compatible with the International Statistical Classification of Diseases and Related Health Problems 10th Revision (ICD-10); length of stay; costs; procedures; and prefecture where the hospital is located, among others. Data for both in- and outpatients were extracted from April 2009 through March 2019.

The MHLW permitted our use of the NDB. The study was approved by the Ethics Committee of Medical Research, University of Occupational and Environmental Health, Japan (approval number: H30-124).

### Definition of mitochondrial diseases

The following diagnoses were defined as mitochondrial diseases: Pearson syndrome (compatible ICD-10 code: D640); pyruvate dehydrogenase complex (PDHC) deficiency (E744); mitochondrial disorders (E888); MELAS (E888); myoclonus epilepsy associated with ragged-red fibers (MERRF) (E888); mitochondrial neurogastrointestinal encephalopathy (MNGIE) (E888); mitochondrial cardiomyopathy (E888); mitochondrial hepatopathy (E888); mitochondrial diabetes (E888); Leigh syndrome (LS) (G318); Alpers’ syndrome (G318); mitochondrial encephalomyopathy (G713); mitochondrial myopathy (G713); Leber’s hereditary optic neuropathy (LHON) (H472); chronic progressive external ophthalmoplegia (CPEO) (H494); and Kearns-Sayre syndrome (KSS) (H498).

These definitions were determined by a well-trained physician and a researcher specializing in mitochondrial diseases. In sampling for this study, patients who had primary or other diagnostic positions were included, while suspected cases were excluded.

### Estimation of the number of patients

The primary outcome of this study was the number of patients with mitochondrial diseases from April 2018 through March 2019. We identified affected individuals using one of the unique identifiers generated by the MHLW and assigned to individuals in NDB. This identifier consists of the health insurance number, date of birth, and sex.

From this data, we also estimated the prevalence of mitochondrial diseases. As the severity of mitochondrial diseases varies widely among patients, and some patients undergo outpatient examination only, we limited our analysis to patients who experienced at least one episode of inpatient care as representative of standard cases requiring clinical intervention above a certain level.

We calculated the prevalence of mitochondrial diseases in Japan by dividing the number of patients with mitochondrial diseases from April 2018 through March 2019 by the total population of Japan as of October 1, 2018^25^ as follows:

$$\text{prevalence}\;(\text{per }100,000\text{ general population})=\frac{(\text{number of patients from April }2018\text{ to March }2019)\times 100,000}{\text{general population on October }1\text{, }2018}$$

We calculated the prevalence of mitochondrial diseases in each prefecture in a similar manner. Further, 95% confidence intervals (CIs) of prevalence were calculated using the Wald method. We also used the Wald method to calculate the 95% CI of the ratio of female patients in Japan.

Additionally, we estimated the standardized prevalence ratio (SPR) of patients in each prefecture using indirect standardization. SPR is defined as follows:

$$\begin{array}{rl} & SPRi=\frac{Oi}{Ei}\times 100\\ & Oi:\;\text{Observed number of patients in i prefecture},\\ & Ei:\;\text{Expected number of patients in i prefecture}\\ & \;=\sum \left\{\begin{array}{c} \left(\text{prevalence of patients in Japan by age class}\right)\\ \times \;(\text{population by age class in i prefecture}) \end{array}\right\} \end{array}$$

The SPR of *i* prefecture is obtained by dividing *Oi*, the observed number of patients, by *Ei*, the expected number of patients. To calculate *Ei*, we used the prevalence in Japan as a reference population, and adjusted for age categorized into three age classes (0–14, 15–64, or ≥65 years old). The 95% CIs of SPRs were estimated using Fisher’s exact CI. For example, a prefecture with an SPR of 100 has the same prevalence as Japan overall, while one with an SPR smaller than 100 has a smaller prevalence than Japan, and vice versa.

We also calculated the empirical Bayes estimator of standardized prevalence ratio (EBSPR) of each prefecture. EBSPR is defined as follows:

$$\begin{array}{rl} & EBSPRi=\frac{Oi+\beta }{Ei+\alpha }\times 100\\ & \alpha ,\beta :\;\text{estimator} \end{array}$$

We estimated the EBSPR using a Poisson-Gamma model.^26^ We expect that use of EBSPR should smooth out the influence of different population sizes in each prefecture on SPR.

Data analyses were performed using Stata 16.0 (StataCorp, College Station, TX, USA) and EB estimator for Poisson-Gamma model Version 2.1.^27^

## RESULTS

### Number of patients and prevalence of mitochondrial diseases

Within the study period, there were fewer male patients with mitochondrial diseases than female patients (47 vs 53; 95% CI for female ratio, 0.51–0.54), and the majority of patients fell within the age class 0–9 years old (Table 1). A total of 3,629 patients were diagnosed with mitochondrial diseases from April 2018 through March 2019, at a prevalence of 2.9 (95% CI, 2.8–3.0) per 100,000 general population.

**Table 1.** Patients’ background (*n* = 3,629)^a^

	*n*	%
Sex, male, *n* (%)	1,712	47

Age class, years, *n* (%)		
0–4	315	9
5–9	333	9
10–14	233	6
15–19	244	7
20–29	352	10
30–39	432	12
40–49	496	14
50–59	431	12
60–69	377	10
70–79	302	8
80 over	114	3

^a^This table shows the number of patients with mitochondrial diseases from April 2018 to March 2019 identified in this study. Median age class was 30–39 years (interquartile range: 15–19, 50–59 years).

Table 2 lists the diagnosis codes and the corresponding number of patients. The majority of patients had diagnosis codes for mitochondrial encephalomyopathy and mitochondrial disorders (1,786 and 1,370, respectively).

**Table 2.** Number of patients with mitochondrial diseases with each diagnosis^a^

ICD-10	Diagnosis code	Diagnosis	Total		Female	

			*n*	%	*n*	%
D640	8846217	Pearson syndrome	—	—	—	—
E744	8848412	PDHC deficiency	81	2	56	69
E888	8845613	Mitochondrial disorders	1,370	38	750	55
E888	8846079	MELAS	284	8	159	56
E888	8846080	MERRF	15	1	—	—
E888	8846084	MNGIE	—	—	—	—
E888	8846224	Mitochondrial cardiomyopathy	174	5	86	49
E888	8846972	Mitochondrial hepatopathy	32	1	15	47
E888	8849469	Mitochondrial diabetes	62	2	39	63
E888	8849470	Mitochondrial diabetes with eye problems	—	—	—	—
E888	8849471	Mitochondrial diabetes with ketoacidosis	—	—	—	—
E888	8849472	Mitochondrial diabetes with coma	—	—	—	—
E888	8849473	Mitochondrial diabetes with neurologic symptom	—	—	—	—
E888	8849474	Mitochondrial diabetes with renal complication	—	—	—	—
E888	8849475	Mitochondrial diabetes with multiple diabetic complications	—	—	—	—
E888	8849476	Mitochondrial diabetes without diabetic complication	—	—	—	—
E888	8849477	Mitochondrial diabetes with diabetic complication	—	—	—	—
E888	8849478	Mitochondrial diabetes with peripheral circulatory disorder	—	—	—	—
G318	8840933	Leigh syndrome	212	6	108	51
G318	8842457	Alpers’ syndrome	—	—	—	—
G713	8841409	Mitochondrial myopathy	253	7	124	49
G713	8841410	Mitochondrial encephalomyopathy	1,786	49	932	52
H472	8848684	LHON	71	2	17	24
H494	8846059	CPEO	106	3	50	47
H498	8831018	Kearns-Sayre syndrome	19	1	10	53

CPEO, chronic progressive external ophthalmoplegia; ICD-10, International Statistical Classification of Diseases and Related Health Problems 10th Revision; LHON, Leber’s hereditary optic neuropathy; MELAS, mitochondrial myopathy, encephalopathy, lactic acidosis, and stroke-like episodes; MERRF, myoclonus epilepsy associated with ragged-red fibers; MNGIE, mitochondrial neurogastrointestinal encephalopathy; PDHC, pyruvate dehydrogenase complex.

^a^This table shows the number of patients with mitochondrial diseases with each diagnosis categorized by domestic diagnosis codes for healthcare claims in Japan. According to the rules for publication of NDB data, we did not show the number of cases in categories with less than 10 patients (indicated by “—” in the table). The sum of patients is not equal to the total number of patients because some patients are given two or more diagnoses.

We also compared the number of patients identified as having mitochondrial diseases in this study with the number using the Japanese medical expense subsidy system, as reported by the government.^28^ The number of patients identified in this study was more than two times greater than the number using the medical expense subsidy system (3,629 vs 1,504).

### Number of patients and prevalence of mitochondrial diseases in each prefecture

**Figure 1.**
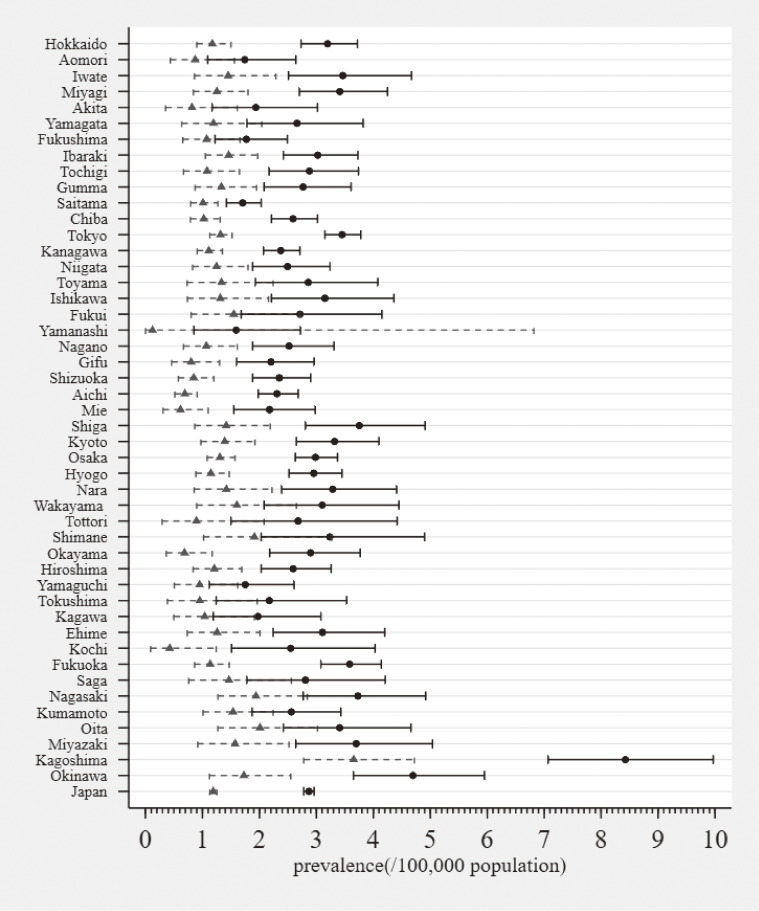
Estimated prevalence of mitochondrial diseases in each prefecture in Japan according to the NDB and government report. Prefectures in this figure are listed according to their geographic location from north-east to south-west. Black points represent the prevalence estimated by this study; solid black lines with caps on both ends represent 95% confidence intervals of the prevalence estimated by this study; grey triangles represent the prevalence indicated by the Japanese government; grey dashed lines with caps on both ends represent 95% confidence intervals of the prevalence indicated by the Japanese government.

Table 3 and Figure 1 show the number of patients and prevalence of mitochondrial diseases in each prefecture from April 2018 through March 2019 in Japan. The prefecture with the greatest number of patients with mitochondrial diseases was Tokyo (*n* = 477/3,629, approx. 13%) while Yamanashi had the fewest (*n* = 13/3,629, approx. 1%). The prevalence of mitochondrial diseases was highest in Kagoshima (8.4/100,000) and lowest in Yamanashi (1.6/100,000).

**Table 3.** Number of patients and prevalence of mitochondrial diseases in each prefecture in Japan from April 2018 to March 2019^a^

	NDB				Government report			
	
Prefecture	*n*	%	Prevalence	95% CI	*n*	%	Prevalence	95% CI
Hokkaido	169	5	3.2	2.7–3.7	62	4	1.2	0.9–1.5
Aomori	22	1	1.7	1.1–2.6	11	1	0.9	0.4–1.6
Iwate	43	1	3.5	2.5–4.7	18	1	1.5	0.9–2.3
Miyagi	79	2	3.4	2.7–4.3	29	2	1.3	0.8–1.8
Akita	19	1	1.9	1.2–3	8	1	0.8	0.4–1.6
Yamagata	29	1	2.7	1.8–3.8	13	1	1.2	0.6–2.0
Fukushima	33	1	1.8	1.2–2.5	20	1	1.1	0.7–1.7
Ibaraki	87	2	3.0	2.4–3.7	42	3	1.5	1.1–2.0
Tochigi	56	2	2.9	2.2–3.7	21	1	1.1	0.7–1.7
Gumma	54	1	2.8	2.1–3.6	26	2	1.3	0.9–2.0
Saitama	125	3	1.7	1.4–2	74	5	1.0	0.8–1.3
Chiba	162	4	2.6	2.2–3	64	4	1.0	0.8–1.3
Tokyo	477	13	3.5	3.2–3.8	182	12	1.3	1.1–1.5
Kanagawa	218	6	2.4	2.1–2.7	102	7	1.1	0.9–1.4
Niigata	56	2	2.5	1.9–3.2	28	2	1.2	0.8–1.8
Toyama	30	1	2.9	1.9–4.1	14	1	1.3	0.7–2.2
Ishikawa	36	1	3.1	2.2–4.4	15	1	1.3	0.7–2.2
Fukui	21	1	2.7	1.7–4.2	12	1	1.6	0.8–2.7
Yamanashi	13	1	1.6	0.8–2.7	1	1	0.1	0.1–6.8
Nagano	52	1	2.5	1.9–3.3	22	1	1.1	0.7–1.6
Gifu	44	1	2.2	1.6–3	16	1	0.8	0.5–1.3
Shizuoka	86	2	2.4	1.9–2.9	31	2	0.8	0.6–1.2
Aichi	174	5	2.3	2–2.7	52	3	0.7	0.5–0.9
Mie	39	1	2.2	1.6–3	11	1	0.6	0.3–1.1
Shiga	53	1	3.8	2.8–4.9	20	1	1.4	0.9–2.2
Kyoto	86	2	3.3	2.7–4.1	36	2	1.4	1.0–1.9
Osaka	263	7	3.0	2.6–3.4	115	8	1.3	1.1–1.6
Hyogo	162	4	3.0	2.5–3.5	63	4	1.1	0.9–1.5
Nara	44	1	3.3	2.4–4.4	19	1	1.4	0.9–2.2
Wakayama	29	1	3.1	2.1–4.5	15	1	1.6	0.9–2.7
Tottori	15	1	2.7	1.5–4.4	5	1	0.9	0.3–2.1
Shimane	22	1	3.2	2–4.9	13	1	1.9	1.0–3.3
Okayama	55	2	2.9	2.2–3.8	13	1	0.7	0.4–1.2
Hiroshima	73	2	2.6	2–3.3	34	2	1.2	0.8–1.7
Yamaguchi	24	1	1.8	1.1–2.6	13	1	0.9	0.5–1.6
Tokushima	16	1	2.2	1.2–3.5	7	1	1.0	0.4–2.0
Kagawa	19	1	2.0	1.2–3.1	10	1	1.0	0.5–1.9
Ehime	42	1	3.1	2.2–4.2	17	1	1.3	0.7–2.0
Kochi	18	1	2.5	1.5–4	3	1	0.4	0.1–1.2
Fukuoka	183	5	3.6	3.1–4.1	58	4	1.1	0.9–1.5
Saga	23	1	2.8	1.8–4.2	12	1	1.5	0.8–2.6
Nagasaki	50	1	3.7	2.8–4.9	26	2	1.9	1.3–2.8
Kumamoto	45	1	2.6	1.9–3.4	27	2	1.5	1.0–2.2
Oita	39	1	3.4	2.4–4.7	23	2	2.0	1.3–3.0
Miyazaki	40	1	3.7	2.6–5	17	1	1.6	0.9–2.5
Kagoshima	136	4	8.4	7.1–10	59	4	3.7	2.8–4.7
Okinawa	68	2	4.7	3.7–6	25	2	1.7	1.1–2.6
	
Japan	3,629	—	2.9	2.8–3	1,504	—	1.2	1.1–1.3

CI, confidence interval; NDB, National Database.

^a^This table shows the number of patients and the prevalence of mitochondrial diseases from April 2018 to March 2019 identified in this study. The same parameters reported by the government based on the number of patients using the Japanese medical expenses subsidy for intractable diseases including mitochondrial diseases are shown as a reference. Prefectures are listed according to their geographic location from north-east to south-west. % values do not sum to 100% because of rounding. Prevalence is prevalence per 100,000 population in each prefecture.

We also compared the number of patients identified as having mitochondrial diseases in each prefecture in this study with the number using the Japanese medical expense subsidy system, as reported by the government.^28^ The number of patients identified in this study was greater than the number using the medical expense subsidy system in all prefectures in Japan.

### SPR and EBSPR of patients with mitochondrial diseases in each prefecture

Table 4 shows the SPRs and EBSPRs of patients with mitochondrial diseases in each prefecture. Similar to the prevalence shown in Table 3, Kagoshima and Okinawa had the highest SPRs of all prefectures, at 294 and 152.3, respectively. In contrast, Yamanashi and Saitama had the lowest SPRs of all prefectures, at 56 and 59, respectively. Although it is important to consider the effect of population size in each prefecture, in Ishikawa and Okinawa, there were large differences in SPR by sex. The SPRs of female and male patients were 75 and 148.8 in Ishikawa, and 183.6 and 117.7 in Okinawa, respectively.

**Table 4.** SPRs and EBSPRs of mitochondrial diseases in each prefecture in Japan from April 2018 to March 2019^a^

	Total				Female				Male			

Prefecture	*n*	SPR	95% CI	EBSPR	*n*	SPR	95% CI	EBSPR	*n*	SPR	95% CI	EBSPR
Hokkaido	169	114.3	97.7–132.9	113.2	96	119.7	96.9–146.1	116.9	73	107.6	84.3–135.3	106.6
Aomori	22	62.6	39.3–94.8	74.2	—	—	—	—	—	—	—	—
Iwate	43	124.1	89.8–167.1	117.4	23	125.1	79.3–187.8	114.7	20	122.8	75–189.6	112.5
Miyagi	79	119	94.3–148.4	115.9	43	123.0	89–165.6	116.6	36	114.5	80.2–158.5	110.3
Akita	19	71.4	43–111.5	82.2	—	—	—	—	—	—	—	—
Yamagata	29	95	63.6–136.4	97.2	15	92.4	51.7–152.4	96.5	14	97.7	53.4–163.9	100.1
Fukushima	33	62.7	43.2–88.1	71.3	18	66.0	39.1–104.2	78.0	15	59.4	33.3–98	76.1
Ibaraki	87	105.7	84.7–130.4	105.1	39	91.7	65.2–125.3	94.0	48	121.1	89.3–160.5	115.6
Tochigi	56	100.1	75.6–130	100.4	29	100.1	67–143.8	100.4	27	100.3	66.1–145.9	101
Gumma	54	96.9	72.8–126.5	97.9	28	96.6	64.2–139.6	98.1	26	97.6	63.8–143	99.3
Saitama	125	59	49.1–70.3	61.8	67	61.1	47.4–77.6	65.7	58	57	43.3–73.6	63.2
Chiba	162	90.2	76.9–105.3	91.1	76	81.4	64.1–101.8	84.0	86	100.1	80–123.6	100.4
Tokyo	477	119	108.6–130.2	118.4	258	122.7	108.2–138.6	121.3	219	115.1	100.3–131.4	114.1
Kanagawa	218	81.9	71.4–93.5	82.9	115	83.4	68.8–100	85.0	103	80.4	65.7–97.6	82.9
Niigata	56	88.7	67–115.2	91.2	31	93.0	63.2–132	95.4	25	84	54.3–124	90.4
Toyama	30	101.6	68.5–145	101.7	13	83.5	44.5–142.9	91.9	17	121.8	70.9–194.9	111.3
Ishikawa	36	109.8	76.9–152	107.3	13	75.0	39.9–128.2	86.7	23	148.8	94.3–223.3	125
Fukui	21	94.8	58.6–144.8	97.6	11	94.1	47–168.4	97.9	10	95.5	45.8–175.6	99.6
Yamanashi	13	56	29.8–95.8	73.8	—	—	—	—	—	—	—	—
Nagano	52	88.8	66.3–116.5	91.4	35	113.7	79.2–158.2	109.8	17	61.3	35.7–98.2	76.4
Gifu	44	76.9	55.9–103.3	82.0	25	82.6	53.4–121.9	88.5	19	70.6	42.5–110.2	82.4
Shizuoka	86	82.1	65.7–101.4	84.5	48	87.8	64.7–116.4	90.5	38	76	53.8–104.3	82.4
Aichi	174	78.6	67.3–91.2	80.0	97	84.8	68.8–103.5	86.6	77	72	56.8–90	76
Mie	39	76.1	54.1–104	81.8	19	70.4	42.4–109.9	81.0	20	82.5	50.4–127.5	90.4
Shiga	53	127.3	95.4–166.5	120.7	28	128.8	85.6–186.1	117.8	25	125.8	81.4–185.7	115.2
Kyoto	86	116.5	93.2–143.9	114.1	40	101.0	72.2–137.5	101.0	46	134.2	98.2–179	124
Osaka	263	103.9	91.7–117.3	103.8	136	100.4	84.2–118.8	100.5	127	107.8	89.9–128.3	107.1
Hyogo	162	102.9	87.7–120	102.8	77	90.9	71.8–113.6	92.4	85	116.6	93.1–144.1	114
Nara	44	116.1	84.4–155.9	112.1	19	92.0	55.4–143.6	95.7	25	144	93.2–212.5	123.9
Wakayama	29	110.6	74.1–158.9	107.5	15	105.6	59.1–174.2	103.4	14	116.3	63.6–195.2	108.2
Tottori	15	94	52.6–155	97.7	—	—	—	—	—	—	—	—
Shimane	22	115	72.1–174.1	109.3	10	98.9	47.5–182	100.2	12	133.5	69–233.2	113.4
Okayama	55	101.3	76.3–131.8	101.4	33	114.2	78.6–160.4	109.9	22	86.5	54.2–130.9	92.6
Hiroshima	73	89.9	70.5–113.1	91.7	34	79.2	54.8–110.7	84.6	39	101.9	72.4–139.3	102
Yamaguchi	24	62.7	40.2–93.3	73.5	11	53.4	26.7–95.6	72.9	13	73.2	39–125.1	87
Tokushima	16	77.9	44.6–126.6	87.9	—	—	—	—	—	—	—	—
Kagawa	19	69.6	41.9–108.7	80.8	—	—	—	—	—	—	—	—
Ehime	42	110.4	79.6–149.2	108.0	23	112.2	71.1–168.3	107.7	19	108.3	65.2–169	105.4
Kochi	18	92.1	54.6–145.6	96.3	—	—	—	—	—	—	—	—
Fukuoka	183	123.3	106.1–142.5	121.4	99	123.5	100.4–150.4	120.2	84	122.7	97.9–151.9	118.8
Saga	23	96.9	61.4–145.4	98.8	12	94.2	48.7–164.5	97.8	11	100.6	50.2–180	101.6
Nagasaki	50	131.2	97.4–172.9	123.0	25	121.2	78.5–179	113.0	25	142.5	92.2–210.3	123.2
Kumamoto	45	89	64.9–119.1	91.9	20	73.2	44.7–113	82.7	25	107.4	69.5–158.5	105.3
Oita	39	120.7	85.8–164.9	114.8	25	143.6	92.9–212	124.5	14	93.6	51.2–157.2	98.1
Miyazaki	40	129.1	92.2–175.8	120.3	19	113.2	68.2–176.8	107.7	21	147.5	91.3–225.4	123.4
Kagoshima	136	294	246.6–347.7	247.7	79	314.2	248.8–391.6	237.3	57	268	203–347.3	196.5
Okinawa	68	152.3	118.3–193.1	139.8	43	183.6	132.9–247.3	152.4	25	117.7	76.2–173.8	111

Japan	3,629	100	96.8–103.3	100	1,917	100.0	95.6–104.6	100	1,712	100	95.3–104.9	100

CI, confidence interval of SPR; EBSPR, empirical Bayes estimator of standardized prevalence ratio; SPR, standardized prevalence ratio.

^a^This table shows SPRs and EBSPRs of patients with mitochondrial diseases in each prefecture from April 2018 to March 2019 identified in this study. SPRs and EBSPRs were calculated for total, female and male patients. According to the rules for publication of NDB data, we did not show the number of cases in categories with less than 10 patients (indicated by “—” in the table). Values were also concealed when the number of either of males or females was less than 10.

EBSPRs in each prefecture provided more conservative results than normal SPRs, indicating that values were reaching closer to 100. Similar to results for normal SPRs, Kagoshima and Okinawa had the highest EBSPRs of all prefectures, at 247.7 and 139.8, respectively. In contrast, Saitama and Fukushima had the lowest EBSPRs of all prefectures, at 61.8 and 71.3, respectively.

## DISCUSSION

Using data from the Japanese NDB, we estimated that the number of patients with mitochondrial diseases from April 2018 through March 2019 was 3,629, with a prevalence of 2.9 per 100,000 general population. This study is the first to comprehensively estimate the number of patients with mitochondrial diseases in Japan, along with the distribution of patients by sex, age, and geographic characteristics using health care claims data from the past 10 years.

The Japanese government has established a medical expense subsidy system for patients with intractable diseases, including mitochondrial diseases. According to government statistics from 2018,^28^ 1,504 patients with mitochondrial diseases used this system. This number is less than half the number of patients with mitochondrial diseases identified in this study (*n* = 3,629). Similarly, the number and prevalence of patients in each prefecture identified as having mitochondrial diseases in this study were also greater than those using the subsidy system. However, it may not be appropriate to compare the number of patients identified in the present study with that in the government report. This is because, while certification for the government subsidy system is typically based on Japanese clinical criteria,^29^ some patients with relatively mild disease severity may not use this system, whose main purpose is to provide treatment-related financial support to patients. Therefore, it may be that the government-reported number of patients will inevitably be an underestimate compared to that identified in the present study.

A previous study in Japan reported that 233 patients had MELAS, with a prevalence of 0.18 (95% CI, 0.17–0.19) per 100,000 general population.^18^ In this study, we identified 284 patients with MELAS, with a prevalence of 0.22 (95% CI, 0.20–0.25) per 100,000 general population. Therefore, the number of patients and prevalence of MELAS identified in this study are comparable to those of the previous study. We expect that this epidemiological information will contribute to improving the health care system for patients with mitochondrial diseases in Japan.

We found that prevalence of mitochondrial diseases differed among prefectures. While the reasons for this are unclear, previous studies from other countries suggest that prevalence may differ by geography.^4^^,^^6^^,^^11^^,^^19^ For example, a study conducted in the northeast of England reported that patients showing clinical manifestations had a prevalence of 9.6 per 100,000 adult general population,^6^ which is about three times higher than that found in this study (2.9 per 100,000 general population). Moreover, a study in Finland suggested that geographical and cultural isolation may cause differences in prevalence.^11^^,^^19^ Furthermore, a study in Australia showed that the prevalence of mitochondrial diseases among children whose mothers were born in Lebanon is much higher than that for mothers born in other countries.^12^ However, we were careful when comparing results obtained using different methods for two main reasons. First, prevalence estimated by a single expert clinical and laboratory referral center can be a greater overestimation than that determined by a study using national health database with large samples. Second, patients extracted from NDB differ in diagnosis period. This difference may affect the number of patients between studies because clinical criteria and the diagnostic methods applied depend on the diagnosis period.^30^

To our knowledge, however, no meaningful report from Asian countries has appeared to date. Further studies are needed to examine the prevalence of mitochondrial diseases in Asia and to compare the results obtained in this study with those in other Asian countries.

We found differences in the prevalence of mitochondrial diseases among prefectures, despite the fact that the genetic background of the Japanese population is thought to be relatively uniform across the country. In addition to genetic factors, the variability in prevalence may be related to differences in health care infrastructure among prefectures in Japan. That is, there may be a concentration of patients in specific medical facilities that are well equipped for diagnosis and treatment. For example, within the Tokyo metropolitan area, the number of patients and prevalence of mitochondrial diseases in Saitama (125, 1.7/100,000), a densely populated prefecture with a population of seven million, was lower than in other large prefectures like Chiba (162, 2.6/100,000) and Kanagawa (218, 2.4/100,000), which are also adjacent to Tokyo. We speculate that there are two main reasons for this. First, there are fewer physicians and specialists per prefecture population in Saitama than Chiba and Kanagawa. Additionally, there are few large hospitals with the ability to provide care for patients with mitochondrial diseases. Second, residents of Saitama have easy access to large hospitals in Tokyo. A similar phenomenon is thought to be occurring in local cities outside the metropolitan area. To improve the provision of health care for patients with mitochondrial diseases, we suggest that, in addition to training healthcare workers and improving clinical guidelines, there is a need to increase the number of medical facilities with the competency to provide care for patients with mitochondrial diseases. Additionally, geographic factors may also be important. We found that patients in Kagoshima and Okinawa had higher prevalence, SPRs, and EBSPRs than all other prefectures. While the reason for the higher values is unclear, these findings suggest that there may be geographic effects because Kagoshima and Okinawa are located in the southernmost part of Japan.

This study has several limitations. First, it is possible that some patients included in this study did not actually have mitochondrial diseases. While a definitive diagnosis of mitochondrial diseases requires an integrated approach, including genetic testing, some of these tests are not covered by the current health insurance system in Japan, and the number of medical facilities with the capacity to perform them is limited. Therefore, it is difficult to determine whether the diagnoses used in this study are definitive of mitochondrial diseases. However, we expect that few patients would have been misdiagnosed because, unlike common or frequent diseases, mitochondrial diseases are carefully and strictly diagnosed by clinicians. Second, due to the nature of the NDB, patients can be duplicated if they or their caregivers change their health insurance scheme due to a job change, unemployment, or employment, thus leading to a potential overestimation of cases. A previous study using the NDB examined the effects of changing health insurance schemes on the estimation of prevalence.^31^ Using the method described in this previous study,^31^ we calculated the maximum impact of changing health insurance schemes to be about 6.6% by summing the proportion of patients who were newly unemployed within a year (1.7%) and the proportion who underwent a job change (4.9%) in 2018.^32^ Therefore, the effect of changing health insurance schemes is likely relatively small. Third, information on patients who receive public assistance is not included in the NDB. The proportion of people who received public assistance in Japan was about 1.65% in February 2019.^33^ Thus, this is expected to have caused only a small underestimation. Despite the above-mentioned limitations, our study provides a valid estimation of the number of patients and prevalence of mitochondrial diseases in Japan.
